# Solidification, remediation and long-term stability of heavy metal contaminated soil under the background of sustainable development

**DOI:** 10.1038/s41598-022-14122-z

**Published:** 2022-06-20

**Authors:** Yuanyuan Li, Shibo Jia, Jiang Liu

**Affiliations:** 1 School of Civil Engineering, Qingdao University of Technology, Qingdao, 266520 Shandong China; 2 Department of Civil and Architectural Engineering, Qingdao University of Technology (School of Linyi), Linyi, 273400 Shandong China

**Keywords:** Environmental sciences, Environmental social sciences

## Abstract

At present, the global pollution has seriously exceeded the standard. With the passage of time, pollution has gradually affected people’s daily lives, but the solution to pollution is far from achieving a better treatment effect. For the treatment of pollution, in addition to considering the treatment effect, it is also necessary to consider whether the treatment method will cause pollution and the cost of the treatment of the pollutants. As one of the lifelines of human survival, the land is also suffering from pollution. The impact of heavy metal pollution is particularly serious, and there is no better solution. Based on this, this paper proposes a curing agent based on sustainable remediation to solve the soil pollution of heavy metals. The main material is Basic oxygen furnace slag (BOFS), which has excellent social development characteristics in all aspects, and the raw materials are calcium carbide residue (CCR) and phosphogypsum (PG) to explore a more suitable curing agent. (consisting of BOFS, CCR, and PG, abbreviated as BCP). The experimental results in this paper show that the volume of pores and pores in the agglomerates are slightly reduced, and the content of curing agent is increased from 4 to 10%, while the corresponding volume is only reduced by 0.006 and 0.017 mL/g. Therefore, it can be seen that the reduction of the pore volume between the aggregates of the stabilized species of BCP has made a major contribution to the strength development.

## Introduction

Globally, more than 20 million hectares of land are contaminated with heavy metal-like substances, with current soil concentrations above geographic baseline or regulatory levels. In situ and ex situ remediation technologies have been developed to remediate heavy metal contaminated sites, including surface capping, electro-extraction, solidification, phytoremediation, and bioremediation. These remediation technologies employ containment, extraction/removal, and immobilization mechanisms to reduce contamination impacts through physical, chemical, biological, electrical, and thermal remediation processes. A comprehensive evaluation indicates that chemical stabilization is an interim soil remediation technique, phytoremediation requires increased efficiency, surface cover and landfilling are suitable for small, heavily contaminated sites, and curing and vitrification are remediation options of last resort. Remediation costs are low. And the cost and duration of soil remediation depends on the technology and the site.

China is the world’s number one industrial country and produces one of the largest volumes of solid waste. What is more, the development of the production economy is accompanied by a substantial growth. Although some of the solid waste is currently disposed of in sanitary landfills, the vast majority of it is disposed of in open piles, which has become a major hidden problem in the process of industrialization and urbanization development. To protect and improve the ecological environment and prevent and control the pollution of the environment by solid waste, the development policy of comprehensive utilization of solid waste is proposed. To further explore the efficient utilization of industrial waste in accordance with China’s national conditions and further promote the utilization of industrial waste in the remediation of heavy metal contaminated soil is an important initiative to achieve efficient resource utilization of industrial waste, and is one of the current research priorities for researchers.

In this paper, the use of industrial waste as the main material for curing agents follows the concept of green and sustainable remediation. The development of sustainable curing agents based on industrial waste as raw materials and the in-depth investigation of the environmental geotechnical properties of industrial waste-based sustainable curing agents for the treatment of industrial heavy metal contaminated soils and the promotion of safe reuse of cured soils or remediation sites have important academic value and have far-reaching economic benefits.

## Related work

Due to the excessive development of industrial activities in the current natural environment, the content of heavy metals in the soil is extremely high, which has become one of the important factors of environmental pollution, and there is no suitable method for its treatment. This has caused many scholars at home and abroad to explore its processing methods. Among them, synthetic hydroxyapatite (HA) is an efficient and environmentally friendly material for the remediation of heavy metal-contaminated soils. However, the application of conventional HA powder in stabilizing contaminated soil is limited due to the high cost of the final product and the difficulty in synthesizing purified HA crystals. Xia W, Y proposed a novel binder called SPC, which consists of single superphosphate (SSP) and calcium oxide (CaO). HA can be formed in the soil matrix through the acid–base reaction between SSP and CaO, resulting in a denser structure and improved mechanical properties of the treated soil. Therefore, SPC can effectively immobilize heavy metals and increase the strength of contaminated soils while keeping the cost relatively low. The results showed that soil pH and unconfined compressive strength (UCS) increased with SPC content and curing time^1^. Heidarzadeh N investigated the unconfined compressive strength (UCS) of common clays and organoclays during curing and stabilization and leaching properties of phenol-contaminated soils. The sample contained 2000 mg/kg of phenol. White cement (15 and 30% by weight [wt%]) was used as a binder, while ordinary clay and organoclays (8, 15, and 30%) were used as additives to reduce the deleterious effects of phenol interfering with cement hydration. The results show that the UCS decreases as the clay amount increases. The UCS values of all samples met the minimum standards for sanitary landfill disposal stipulated by developed countries. Leaching tests showed that the degree of leaching decreased with increasing clay content in all samples of both clay types^2^. Stabilization/solidification (S/S) has been successfully applied to many Superfund sites contaminated with organic materials. However, the long-term effectiveness of this method has not been fully evaluated, and the soil volume increases after treatment, which is not conducive to subsequent disposal. Ma F research developed a new method for S/S of PAHs-contaminated soil facilitated by sulfonated oil (SO). Freeze–thaw durability tests showed that the leaching properties of PAHs were not affected by freeze–thaw cycles. After 12 freeze–thaw cycles, the UCS values of PC-AC-SO-treated soil samples were 2.2–3.4 times higher than those of PC-AC-treated soil samples. The PC-AC-SO treated soil resisted disintegration better than the PC-AC treated soil. SEM micrographs showed that soil compaction was significantly improved after SO treatment^3^. D Wang developed an acidic soil remediation agent (ASRA) in which ASCs were supported by ATP and loaded the resulting ASC-ATP into the micro/nanopores of BCs. ASRA with porous nanonetwork structure can effectively suppress the loss of Ca2+ and increase the pH value of acidic soil. At the same time, it was shown that ASRA was effective on acidic soils in both lateral and vertical directions. Pot experiments proved that ASRA could effectively reduce the absorption of Cr(VI) in maize and had a positive effect on maize height, leaf chlorophyll content, and root length. Therefore, this work may provide a basic and facile method for the remediation of Cr(VI)-pollutants^4^. Lu presented a systematic on-site remediation case involving heavy metals and organic pollutants in the soil and groundwater of a historic industrial site in Shanghai, China. He conducted laboratory-scale experiments and field tests to determine the optimal parameters for removing contaminants from soil and groundwater. Its research found that adding sodium dithionite and ferrous sulfate with a mass content of 3% ± 6% can achieve the goal of remediation of hexavalent chromium in the soil. The results show that it is feasible to use chemical reduction and curing/stabilization methods to carry out on-site and off-site restoration of this site, which can provide a reference for realistic restoration of similar sites^5^. Du X C used TCLP and Tessier sequential method to analyze the solidification effect extraction method. The results show that the addition of ferrous ammonium phosphate can effectively solidify the lead in the contaminated soil. The content of ferrous ammonium phosphate is 1.5%, which can meet the solidification requirements. The greater the amount of curing agent added, the higher the stability efficiency^6^. Sahnoune R mainly focuses on the detailed characterization of 4 soil samples collected near BOULIMAT, a wild garbage dump located 15 km west of the city of Bejaia, Algeria. The samples were characterized by atomic absorption spectroscopy, X-ray diffraction, fluorescence X-ray, and infrared spectroscopy. The data showed high concentrations of metal elements in soil samples, especially Zn (2651.8 mg.kg) and Ni (163.44 mg.kg). For their repair, stabilization/curing (S/S) processes using hydraulic binders seem promising to reduce the fouling capacity of metals. This method greatly reduces the amount of pollutants; the removal rate of Ni is 98% and that of Zn is 99%. XRD analysis technique revealed the presence or absence of metal elements in the crystalline phase^7^. The research results of the above scholars have certain effects on the treatment of heavy metal pollution. However, there is no doubt that these treatment methods can only respond to a specific metal to improve the effect, and the treatment effect of other heavy metals may be somewhat poor and arbitrary. However, the treatment methods of heavy metal pollution are still being explored. It is believed that with the passage of time, better methods will emerge, and in the process, many people will provide them with perfect cornerstones.

## Methods of solidification and remediation of heavy metal contaminated soil under the background of sustainable development

### Overview of curing/stabilization method

The remediation principle of the solidification stabilization method is mainly through the chemical reaction between the solidifying agent and the soil system, so that the heavy metal pollutants can be solidified and stabilized by physical adsorption, chemical absorption, sedimentation, ion exchange, passivation and other methods^8^. At the same time, the addition of the curing agent also provides an alkaline environment for heavy metals and inhibits the migration of heavy metals^9^.

Cement curing mechanism: When the cement is in contact with water, the substances in the cement will rapidly undergo a hydration reaction^10^:

Hydration of Tricalcium Silicate

1 $$ 3{\text{CaO}} \cdot {\text{SiO}}_2 + {\text{nH}}_2{\text{O}} \to 2{\text{CaO}} \cdot {\text{SiO}}_2 \cdot {\text{iH}}_2{\text{O}} + {\text{Ca}}({\text{OH}})_2 $$

2 $$ 3{\text{CaO}} \cdot {\text{SiO}}_2 + {\text{nH}}_2{\text{O}} \to {\text{CaO}} \cdot {\text{SiO}}_2 \cdot {\text{xH}}_2{\text{O}} + 2{\text{Ca}}({\text{OH}})_2 $$

3 $$  2(3{\text{CaO}} \cdot {\text{SiO}}_{{\text{2}}} {\text{)}} + {\text{nH}}_{{\text{2}}} {\text{O}} \to {\text{3CaO}} \cdot {\text{2SiO}}_{{\text{2}}}  \cdot {\text{ iH}}_{2} {\text{O}} + {\text{3Ca}}({\text{OH}})_{{\text{2}}}     $$

4 $$  2(3{\text{CaO}} \cdot {\text{SiO}}_{{\text{2}}} {\text{)}} + {\text{nH}}_{{\text{2}}} {\text{O}} \to {\text{2(CaO}} \cdot {\text{SiO}}_{{\text{2}}}  \cdot {\text{xH}}_{{\text{2}}} {\text{O)}} + {\text{4Ca(OH)}}_{{\text{2}}}  $$

Hydration of Dicalcium Silicate

5 $$ 2{\text{CaO}} \cdot {\text{SiO}}_{2}  + {\text{nH}}_{2} {\text{O}} \to 2{\text{CaO}} \cdot {\text{SiO}}_{2}  \cdot {\text{nH}}_{2} {\text{O}} $$

6 $$  2{\text{CaO}} \cdot {\text{SiO}}_{2}  + {\text{iH}}_{2} {\text{O}} \to {\text{CaO}} \cdot {\text{SiO}}_{2}  \cdot {\text{xH}}_{2} {\text{O}} + {\text{Ca}}({\text{OH}})_{2}   $$

7 $$  2(2{\text{CaO}} \cdot {\text{SiO}}_{2} ) + {\text{nH}}_{2} {\text{O}} \to 3{\text{CaO}} \cdot 2{\text{SiO}}_{2}  \cdot {\text{iH}}_{2} {\text{O}} + {\text{Ca}}({\text{OH}})_{2}  $$

8 $$  2(2{\text{CaO}} \cdot {\text{SiO}}_{2} ) + {\text{nH}}_{2} {\text{O}} \to 2({\text{CaO}} \cdot {\text{SiO}}_{2}  \cdot {\text{iH}}_{2} {\text{O}}) + 2{\text{Ca}}({\text{OH}})_{2}  $$

Hydration of tricalcium aluminate

9 $$   3{\text{CaO}} \cdot {\text{AI}}_{2} {\text{O}}_{3}  + {\text{nH}}_{2} {\text{O}} \to 3{\text{CaO}} \cdot  {\text{AI}}_{2} {\text{O}}_{3}   \cdot {\text{nH}}_{2} {\text{O}} $$

If calcium oxide is present, the hydration reaction is

10 $$  3{\text{CaO}} \cdot {\text{AI}}_{2} {\text{O}}_{3}  + {\text{Ca}}({\text{OH}})_{2}  + {\text{nH}}_{2} {\text{O}} \to 4{\text{CaO}} \cdot {\text{AI}}_{2} {\text{O}}_{3}  \cdot {\text{xH}}_{2} {\text{O}}  $$

Hydration of Tetracalcium Ferroaluminate

11 $$  4{\text{CaO}} \cdot {\text{AI}}_{{\text{2}}} {\text{O3}} \cdot {\text{Fe}}_{{\text{2}}} {\text{O}}_{3}  + {\text{CaSO}}_{4}  \cdot 2{\text{H}}_{2} {\text{O}} + {\text{Ca}}({\text{OH}})_{2}  \to 3{\text{CaO}}({\text{AI}}_{2} {\text{O}}_{3} ,{\text{Fe}}_{2} {\text{O}}_{3} ) \cdot 3{\text{CaSO}}_{4}   $$

When Portland cement comes into contact with polluted soil, it not only reacts with pore water, but also reacts with clay minerals and heavy metal pollutants in polluted soil^11^. This makes the lifting mechanism of Portland cement on heavy metal polluted soil more complicated than that on clean soil (without heavy metal pollutants) treated by Portland cement^12^.

12 $$ {\text{SiO}}_2 + {\text{Ca}}({\text{OH}})_2 + {\text{yH}}_2{\text{O}} \to {\text{CaO}} \cdot {\text{SiO}}_2 \cdot ({\text{y}} + 1){\text{H}}_2{\text{O}} $$

13 $$ {\text{AI}}_{{\text{2}}} {\text{O}}_{{\text{3}}}   + {\text{Ca}}({\text{OH}})_2 + {\text{yH}}_2{\text{O}} \to {\text{CaO}} \cdot {\text{AI}}_2{\text{O}}_3 \cdot ({\text{y}} + 1){\text{H}}_2{\text{O}} $$

In addition to satisfying ion exchange, a large amount of Ca2+ produced by cement hydration will also react with active silica and alumina in clay minerals in an alkaline environment and pozzolanic reaction^13^, as shown in the above equation and generate a certain amount of C–S–H. This part of C–S–H significantly enhanced the adsorption and encapsulation of C–S–H generated in hydrated calcium silicate.

14 $$ {\text{Ca}}({\text{OH}})_2 + {\text{CO}}_2 \to {\text{CaCO}}_3 + {\text{H}}_2{\text{O}} $$

15 $$ 3CaO \cdot 2{\text{SiO}}_2 \cdot 3{\text{H}}_2{\text{O}} + {\text{CO}}_2 \to {\text{CaCO}}_3 + 2({\text{CaO}} \cdot {\text{SiO}}_2 \cdot {\text{H}}_2{\text{O}}) + {\text{H}}_2{\text{O}} $$

16 $$ {\text{CaO}} \cdot {\text{SiO}}_2 \cdot {\text{H}}_2{\text{O}} + {\text{CO}}_2 \to {\text{CaCO}}_3 + {\text{SiO}}_2 + {\text{H}}_2{\text{O}} $$

The cement stone (CH) in the cement hydration product and the Ca2+ dissolved in the pore water react with carbon dioxide (CO2) in the air and carbonate ions (CO3 2-) dissolved in the pore water, respectively, to form calcium carbonate (CaCO3), as shown in the above equation. The generated CaCO3 has the characteristics of cementing soil particles and filling the pores of polluted soil, which further increases the density of polluted soil and promotes the increase of soil strength^14^.

### Application Basis of Curing Agent for Sustainable Repair

1. Demand for green and sustainable restoration

**Figure 1:**
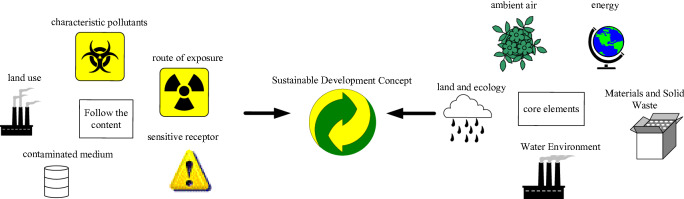
Decision-making perspectives of traditional and green sustainable restoration.

Western developed countries have accumulated rich experience in the practice of dealing with land pollution and site remediation and redevelopment, have established a legislative mechanism based on soil pollution prevention and a sustainable soil environmental protection policy system, and developed a green and sustainable soil environmental restoration management and technology evaluation system, and have put forward a diverse social cooperation mechanism to ensure the safety of regional soil environment and encourage the safe reuse of brown fields^15^. There are significant differences between the decision-making perspectives of traditional site restoration and green sustainable restoration, as shown in Fig. 1.

(2) Application of curing agent

In the early stage of site restoration, a lot of research work has also been done in the screening of curing and stabilization. In the early days, it mainly used the experience of the American Super Fund and used a large number of Portland cement materials. Compared with developed countries such as Europe and the United States, there are significant differences in the site management policies and site pollution of contaminated sites in China^16^. Therefore, in the process of exploration of contaminated site remediation technology, the shortcomings of Portland cement curing and stabilization technology are gradually exposed^17^:

1. The applicability of soil contaminated with high concentrations of heavy metals is extremely poor^18^. A large number of research results have proved that the presence of high concentrations of heavy metals, such as Pb, Zn, Cu, and Cd, in polluted soil can significantly inhibit the reaction of Portland cement hydration products.
2. Cement is an unsustainable material. Cement production is an unsustainable material with high energy consumption and high emissions, which contradicts the concept of green sustainable restoration advocated internationally^19,20^.

Portland cement has obvious drawbacks in the field of heavy metal contaminated site remediation in China. Therefore, it is necessary to actively develop high-efficiency and environment-friendly new curing agents as a substitute for Portland cement and promote the application of new curing agents in site remediation^21^. In addition, based on the concept of green and sustainable restoration, some scientific researchers invest the research and development of curing agents for industrial waste. Since China is a big industrial country, a large amount of industrial waste is produced every year, and using it as a curing agent for the remediation of polluted sites has significant economic, environmental and social benefits^22^.

### Screening of sustainable curing agent raw materials

**Figure 2:**
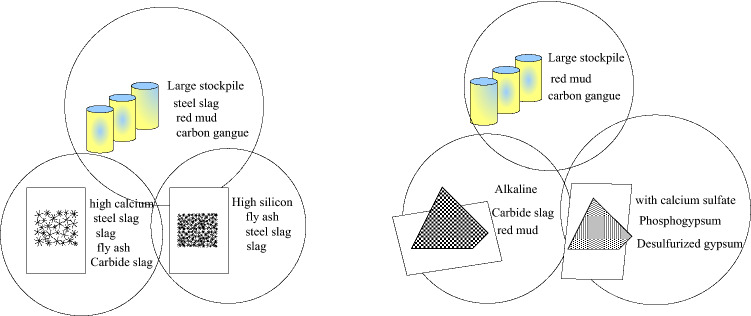
Screening criteria for sustainable curing agent raw materials and excitation materials.

The sustainable curing agent raw material screening includes the main material screening and the excitation material screening,the screening conditions of the curing agent main material and the excitation material are shown in Fig. 2.

**Figure 3:**
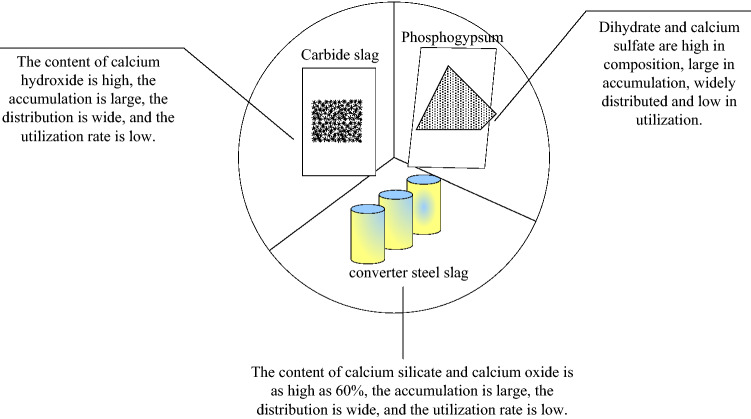
Grouping effects of sustainable curing agents.

Based on China’s industrial structure, converter steel slag is selected as the main material of the curing agent. Studies have shown that the hydration activity of converter steel slag is significantly improved under the excitation of lime and gypsum^23^. In this study, we tried to find industrial wastes containing calcium hydroxide and calcium sulfate dihydrate to replace lime and gypsum, respectively. On the basis of significantly improving the reactivity of converter steel slag, the sustainability of steel slag curing agent was increased. The role of each component of the curing agent is shown in Fig. 3.

**Figure 4:**
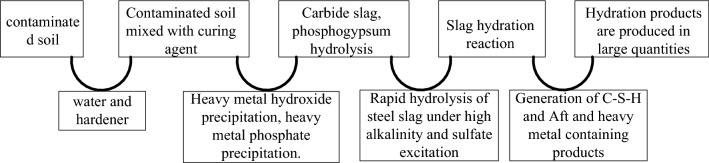
Reaction process of steel slag-based curing agent.

When BCP curing agent was added to the contaminated soil, calcium carbide slag and phosphogypsum were rapidly dissolved in the pore water, and in the pore solution with high pH and high concentration of SO_4_^2+^, the calcium silicate in the converter steel sl was completely depolymerized into simple structural units, and in the pore solution with high concentration of Ca^2+^ and SO_4_^2+^, the simple structural units were condensed into a large amount of gelatinous hydrated calcium silicate (C-SH) with cement formation. Through the combined excitation of calcium carbide slag and phosphogypsum on converter steel slag, the hydration activity of converter steel slag was significantly improved, and the number of hydration products was significantly increased^24^. Combined with the physical and chemical properties of the relevant hydration reaction products, the control mechanism of BCP-solidified heavy metal contaminated soil strength enhancement and heavy metal stabilization is inferred, Fig. 4 shows the reasoning flow for the reaction process of the metal stabilization control mechanism.

## Solidification and remediation of heavy metal contaminated soil under the background of sustainable development

### Test material

1. Heavy metal contaminated soil

The experimental materials were obtained from the relocation legacy of an auto parts manufacturer in The province. In the past few decades, part of the heavy metal waste liquid from production activities has penetrated the foundation soil, and the contaminated foundation soil has been sampled after the ground buildings have been demolished and the concrete ground has been excavated. Due to the presence of construction waste on the surface of the foundation after the floor was removed, we took soil samples 50 cm below the surface. The survey results of pollutants in the site soil are shown in Table 1.Compared with the first type of screening value in the construction land, soil pollution risk management and control standards published by the state the concentration of heavy metals exceeds the control limit.

**Table 1:** Pollutant types and concentrations.

Pollutant item	Site pollutant concentration (mg/kg)	First-class land use screening value (mg/kg)
Ni	21.2 ~ 103,000	150
Zn	32.5 ~ 9126	–
Cd	1.76 ~ 2.17	20
Pb	0.011 ~ 0.638	20
Hg	10.7 ~ 388	8
Cu	0.011 ~ 388	2000
Cr^6+^	62.5 ~ 128	3
1,2-Dichloroethane	0.001 ~ 0.005	0.5
Tetrachloroethylene	0 ~ 0.009	11

It can be found that the concentrations of heavy metals nickel (Ni) and zinc (Zn) in the shallow foundation soil of the site are relatively high, the highest concentrations of heavy metals Ni and Zn can reach 103,000 and 9126 mg/kg, respectively, and the concentrations of the other six heavy metals are relatively low.

Semi-quantitative analysis of the oxide composition and content of the contaminated soil for the test was carried out by X-ray fluorescence spectroscopy (XRF). The test results are shown in Table 2. It is found that the chemical components of the soil used in the test are mainly SiO_2_, Al2O3, and Fe_2_O_3_.

**Table 2:** Chemical composition of contaminated soil (mg/kg).

SiO_2_	AI_2_O_3_	Fe_2_O_3_	K_2_O	MgO	Na_2_O	CaO	TiO_2_	NiO	SO_3_
63.5	16.1	4.8	2.4	2.2	1.3	1.9	0.73	0.7	0.6
ZnO	MnO	CI	BaO	P_2_O_5_	ZrO_2_	SrO	V_2_O_5_	Rb_2_O	Burn vector
0.4	0.1	0.08	0.06	0.03	0.03	0.02	0.01	0.01	4.9

(2) Curing agent

The sustainable curing agent developed in this study consists of three industrial wastes including converter steel slag (BOFS), carbide slag (CCR), and phosphogypsum (PG). The curing agent is named BCP. The mass ratio of converter steel slag (BOFS), calcium carbide slag (CCR), and phosphogypsum (PG) in the curing agent is 6:3:1.

**Figure 5:**
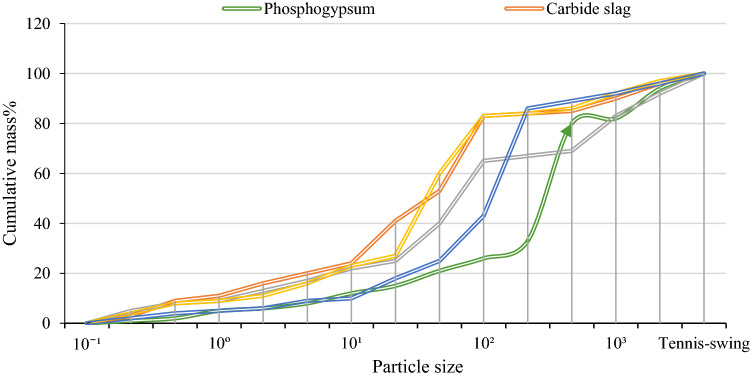
Particle size distribution curves of converter, steel slag, carbide slag, and phosphogypsum.

The specific gravity, pH value, specific surface area (SSA), and particle size distribution of converter, steel slag, carbide slag, and phosphogypsum are shown in Table 3. The particle size distribution curve is shown in Fig. 5.

**Table 3:** Main physicochemical properties of converter steel slag,carbide slag and phosphogypsum.

Grouping	Proportion	pH	SSA (m^2^/kg)	Particle size distribution		
				< 0.005 mm	0.005 ~ 0.075 mm	> 0.075 mm
Steel slag	3.4	11	222	5.6	84.1	10.3
	3.35	11.03	306	16.5	79.42	4
	3.2	11.2	410	22.2	76	1.8
Carbide slag	2.2	12.6	12,628	19.1	73.2	7.7
Phosphogypsum	3	4.3	557	9.1	71.2	19.7

### Experimental method scheme

First, fully dry purchased converter steel slag powder, calcium carbide slag powder, and phosphogypsum powder to a moisture content of less than 1%, and ensure a certain moisture content during the curing process it was 15.6%.

1. Design of the solidification and stabilization effect of steel slag, carbide slag, and phosphogypsum

To explore a better effect of solidification and stability, the research in this paper evaluates the strength of the solidified soil, which is achieved by the unconfined compressive strength, and the stabilization efficiency of heavy metals is also evaluated by the heavy metal stability rate ${\text{HF}}GN$. The calculation equation is as follows:

17 $$ {\text{HF}}GN = \frac{D0GN - DsGN}{{D0GN}} $$

In the equation, the leaching concentration of heavy metals in the table polluted soil is ${\text{D}}oGN$; while ${\text{Ds}}GN$ represents the leaching concentration of heavy metals in the solidified soil; GN refers to the heavy metals Ni and Zn, that is, the stability rates of the two metals can be expressed as ${\text{HF}}Ni$ and ${\text{HFZ}}n$, respectively.

(2) Optimization design of curing agent components

In this study, the comprehensive scoring method^25^ was used to comprehensively consider the effects of each component of BCP curing agent on the improvement of the strength of polluted soil and the stability of heavy metals. Among them, the strength improvement effect is evaluated by relative strength ${\text{P}}iu$, and its calculation method is below.

18 $$ {\text{P}}iu = \frac{Rui}{{Ru\max }} $$

Among them, Ru represents the infinite compressive strength. Ruimax is the maximum intensity value where the relative stability rate ${\text{Q}}i{\text{c}}({\text{Ni)}}$ of heavy metals Ni and the relative stability rate ${\text{Q}}i{\text{c}}({\text{Zn)}}$ of heavy metals Zn are calculated as follows.

$$ {\text{Q}}_{ic} \left( {{\text{Ni}}} \right) = \frac{{{\text{Q}}_{{{\text{ic}}}} \left( {{\text{Ni}}} \right)}}{{Q_{{{\text{umax}}\left( {{\text{Ni}}} \right)}} }} $$

$$ {\text{Q}}_{ic} \left( {{\text{Zn}}} \right) = \frac{{{\text{Q}}_{{{\text{ic}}}} \left( {{\text{Zn}}} \right)}}{{Q_{{{\text{umax}}\left( {{\text{Zn}}} \right)}} }} $$

The power coefficient Bi is calculated as

19 $$ {\text{Bi}} = Piu + Qic(Ni) + Qic(Zn) $$

Among them, in table the orthogonal experimental design sample size, the sample size in this study is 27, namely ${\text{i}} = 1,2, \ldots ,27$.

To ensure the accuracy and repeatability of the test results, in this study, three groups of parallel samples were prepared for unconfined compressive strength test (UCT), leaching toxicity test (LT), pH test (pH) and electrical conductivity test (EC) of contaminated soil samples or solidified soil samples. Then, the average value was calculated from the test results of 3 parallel samples, and the average value of each parameter was used for subsequent analysis and discussion. At the same time, the standard deviation of each parameter value was calculated and marked with error bars in the corresponding graph. In addition, one group of samples were set up for BCR SEP, ANC, MIP, XRD, SEM and EDS tests. The type of curing agent, the amount of curing agent, the curing time, and the number of parallel samples of the test soil samples used are summarized, and the relevant content is shown in Table 4. Among them, the maintenance day here refers to the maintenance time.

**Table 4:** Experimental sample preparation control parameters for different series.

Purpose	Test items	Hardener	Dosage (%)	Moisture content (%)	Dry density (g/cm^3^)	Maintenance days
Evaluation of curing stability of raw materials	LT	BOFS	0、4	21	1.6	3 ~ 180
	UCT	BOFS	0、4	21	1.6	3 ~ 180
	LT	CCR	0、4	21	1.6	3 ~ 180
	UCT	CCR	0、4	21	1.6	3 ~ 180
	LT	PG	0、4	21	1.6	3 ~ 180
	UCT	PG	0、4	21	1.6	3 ~ 180
BCP curing agent component optimization	LT	BCP	0、4	21	1.6	28
	UCT	BCP	0、4	21	1.6	28
Evaluation of BCP curing stability	LT	BCP	0、4、6、8、10	Wopt^g^	0.95	7、14、28、60、90
	UCT	BCP	0、4、6、8、10	Wopt^g^	0.95	7、14、28、60、90
	pH	BCP	0、4、6、8、10	Wopt^g^	0.95	7、14、28、60、90
	EC	BCP	0、4、6、8、10	Wopt^g^	0.95	7、14、28、60、90
Analysis of BCP curing and stabilization mechanism	BCR	BCP	0、4、6、8、10	Wopt^g^	0.95	7、14、28、60、90
	ANC	BCP	0、4、6、8、10	Wopt^g^	0.95	7、28、90
	MIP	BCP	4、6、8、10	Wopt^g^	0.95	7、28、90
	XRD	BCP	8、20、50	–	–	7、28、90
	XRD	BCP	Pure pulp	–	–	28
	SEM	BCP	Pure pulp	–	–	28
	EDS	BCP	Pure pulp	–	–	28

### Optimization test results of curing agent components

**Figure 6:**
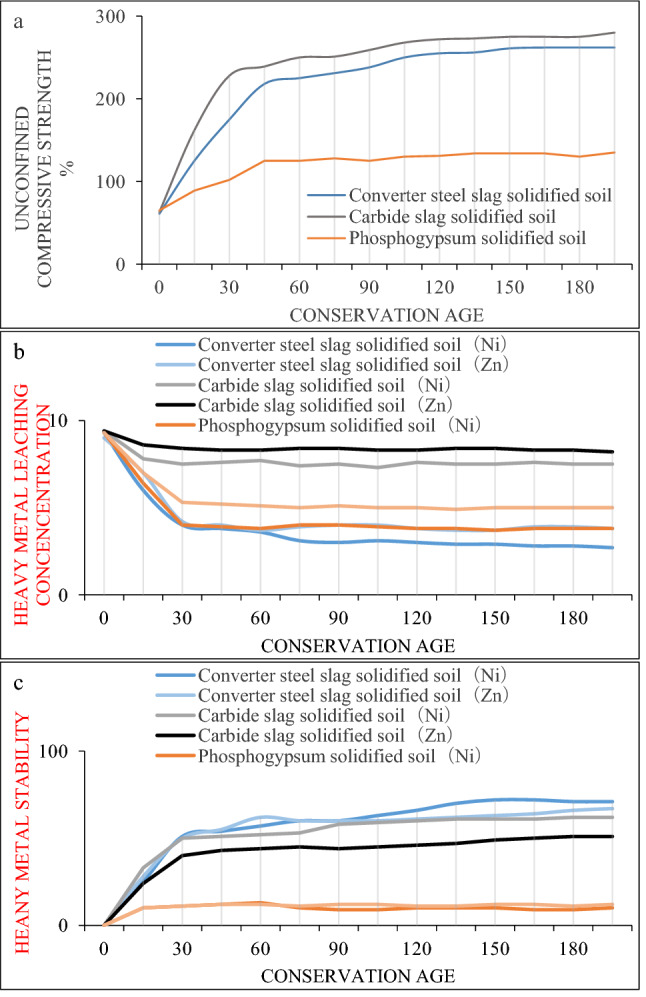
Unconfined compressive strength, heavy metal concentration, and heavy metal stability rate of BCP raw material-solidified soil; (**a**) Unconfined compressive strength of solidified soil; (**b**) Stabilization rate and leaching concentration of heavy metals; (**c**) Stabilization rate of heavy metals.

Firstly, the solidification and stabilization effects of three raw materials (converter steel slag, carbide slag and phosphogypsum) in BCP curing agent on polluted soil were analyzed. Because the infinite lateral compressive strength can evaluate the strength of the solidified soil, it is also used to detect the change of the solidifying agent with the curing time. Therefore, the infinite side compressive strength is used as the y-axis coordinate. Under standard curing conditions, the unconfined compressive strength and heavy metal stability rate of three raw materials, converter steel slag, calcium carbide slag, and phosphogypsum cured soil of curing agent BCP at different curing ages are shown in Fig. 6.

It can be seen from Fig. 6a that the addition of converter steel slag, carbide slag, and phosphogypsum can improve the unconfined compressive strength of polluted soil; and with the extension of curing age, the unconfined compressive strength of the solidified soil increases gradually. And among the three kinds of raw material solidified soil, the unconfined compressive strength of carbide slag solidified soil is the largest, the converter steel slag solidified soil is in the middle, and the phosphogypsum solidified soil is the smallest. The leaching concentrations of heavy metals in converter, steel slag, carbide slag, and phosphogypsum solidified soil are shown in Fig. 6b. The heavy metal leaching concentration in Fig. 6b was calculated according to the formula to obtain the heavy metal stability rate and plotted in Fig. 6c. It can be seen that, similar to the development law of unconfined compressive strength of converter, steel slag, carbide slag, and phosphogypsum solidified soil, the stability rate of the three raw materials to heavy metals increases rapidly in the early curing stage; however, with the increase of the curing age, the growth rate of heavy metal stability gradually slowed down; by the end of the test, the stability rate of heavy metals was basically stable. At the same curing age, the stability rates of heavy metals Ni and Zn in the solidified soil of the three raw materials were slightly different; but with the increase of the curing age, the growth trend was basically the same. During the 28-day curing period and the 180-day curing period of the converter steel slag, carbide slag and phosphogypsum solidified soil, the stability rates of heavy metal Ni were 72, 85 and 75% respectively, while those of heavy metal Zn were 81 and 85 and 92%. In addition, in terms of heavy metal stabilization rate, the heavy metal stabilization rate of converter steel slag is the largest, calcium carbide slag is in the middle, and phosphogypsum is the smallest.

Considering the effect of curing age on the unconfined compressive strength of the solidified soil and the stability rate of heavy metals in the solidified soil, and combining with the BCP curing agent components to optimize the test period, this study set the curing age as 28 days in the three-component optimization test of curing agent.

## Solidification stability analysis of heavy metal contaminated soil

### Environmental geotechnical properties of Bcp-solidified soil

1. Basic soil property parameters of solidified soil

After obtaining the optimal ratio of BCP curing agent, the physicochemical properties of the cured soil with different BCP contents were further analyzed. The cured soil samples with different BCP curing agent contents were prepared, respectively, and the physical and chemical characteristic parameters were tested after curing for 28 days. The test results are shown in Table 5.

**Table 5:** Main parameters of the solidified soil with different BCP contents.

Index		BCP curing agent dosage				
		0%	4%	6%	8%	10%
Proportion		2.7	2.7	2.7	2.7	2.7
pH		5.8	9.3	9.7	10.5	11.2
Cation exchange capacity CEC (cmol/kg)		4.1	6.1	12.8	17.6	23.1
Specific surface area SSA (m^2^/g)		87	84.7	79.8	79.3	65.3
Organic matter content OMC (%)		1.9	1.4	1.2	0.7	0.6
Particle size distribution%	Clay < 0.005 mm	22	20	19	15	6.9
	powder 0.005 ~ 0.075 mm	70	69	63.2	63.1	68
	Gravel 0.075 ~ 2 mm	7.6	11	18	21.6	25

It can be found from Table 5 that: with the increase of the content of BCP curing agent, the organic matter content of the cured soil is significantly reduced. Compared with the contaminated soil, the organic matter content of the 4%BCP and 10%BCP-solidified soils decreased by 0.46 and 1.31%, respectively. The reduction of the organic matter content in the contaminated soil may be due to the oxidizing property of the converter steel slag^26^. The reduction of the organic matter content in the contaminated soil can significantly improve the strength of the silicate-based material to stabilize the soil and weaken the improvement effect of the silicate-based material on the organic matter-containing contaminated soil.

(2) The pH of the solidified soil.

**Figure 7:**
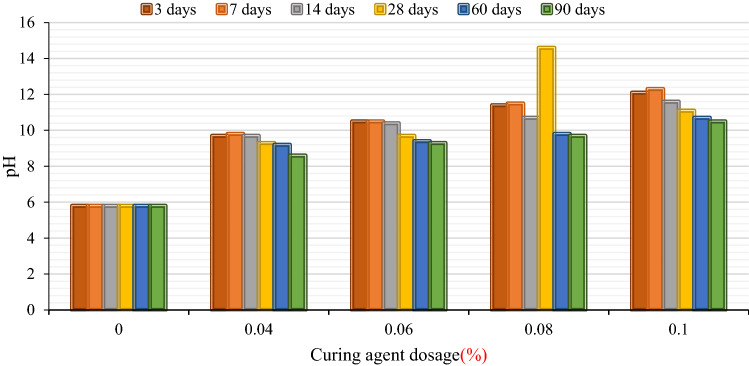
Test results of pH of BCP-stabilized soil.

Figure 7 shows the change rule of pH value of stabilized soil with BCP content and curing age.

It can be seen from Fig. 7 that the contaminated soil (with a curing agent content of 0%) is weakly acidic, and its pH value is 5.8. The main reason is that a large amount of acidic waste liquid infiltrated into the foundation during the production process of the auto parts, resulting in the acidification of the shallow foundation soil of the workshop. When the BCP curing agent was added, the pH value of the cured soil increased significantly and increased significantly with the increase of the curing agent content.

The increase of the pH value of polluted soil by BCP curing agent not only significantly reduces the activity of heavy metal pollutants in the soil, improves the ability of polluted soil to resist external erosion, but also effectively broadens the way of safe reuse of polluted soil. For example, the solidified soil can be used as an engineering filler or the polluted site can be redeveloped as the building foundation, which effectively avoids the safety problem caused by the corrosion and damage of the building by the acidic ions in the polluted soil.

### Strength properties of BCP-stabilized soil

**Figure 8:**
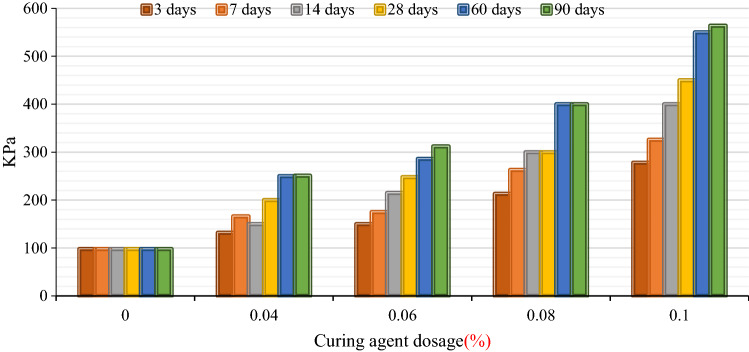
Unconfined compressive strength of BCP-cured soil.

The unconfined compressive strength test results of BCP-cured soil are shown in Fig. 8.

It can also be found from Fig. 8 that with the increase of the BCP curing agent content, the unconfined compressive strength of the cured soil increases gradually. Taking the 14-day curing period as an example, the unconfined compressive strengths of 4, 6, 8, and 10% BCP-solidified soils are 190, 212, 303, and 431 kPa, which are 2.1, 2.4, 3.4 and 4.8 times the unconfined compressive strength of the contaminated soil, respectively. In addition, the unconfined compressive strength of the cured soil increases gradually with the curing age regardless of the BCP curing agent content; however, the strength of the solidified soil increased more rapidly in the early stage of curing, and the strength increased gradually with the increase of curing age.

### Leaching toxicity of BCP-stabilized soil

**Figure 9:**
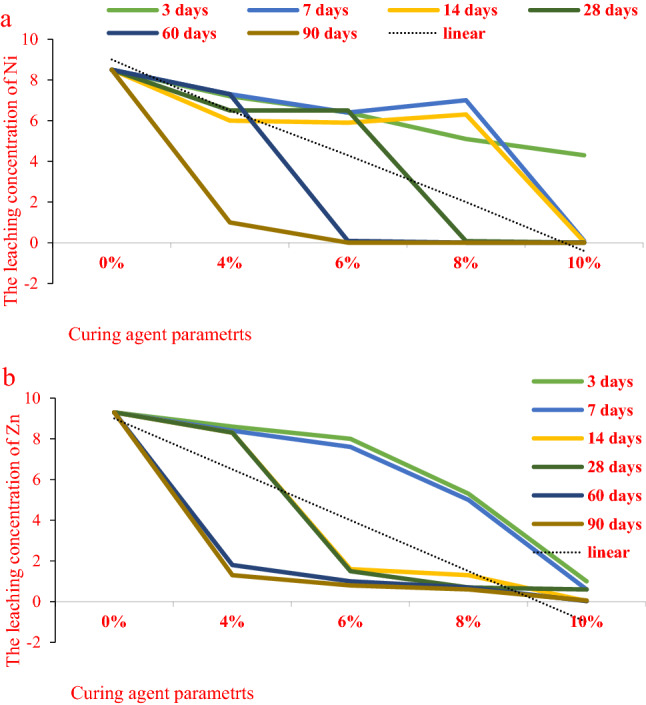
Leaching concentration of heavy metals in BCP-stabilized soil.

Figure 9 shows the test results of heavy metal leaching concentration in the cured soil with different BCP curing agent dosage and curing age.

It can be seen from Fig. 9 that the leaching concentrations of heavy metals Ni and Zn in the polluted soil are 8.5 and 9.3 mg/L, respectively, In the groundwater quality standard for Class IV water, the limits for Ni and Zn are: Ni ≤ 0.10 mg/L, Zn ≤ 5.00 mg/L, and their concentrations are far higher than the Groundwater Quality Standard. It can be seen that the leaching toxicity of the polluted soil is high, and the heavy metals are in higher transport capacity. After the addition of BCP curing agent, the leaching concentration of heavy metals was significantly reduced. For example, the leaching concentrations of heavy metals Ni and Zn in the solidified soil with BCP curing agent content of 4% were 1.06–6.61 mg/L and 1.11–7.11 mg/L, respectively. With the increase of curing agent content, the leaching concentration of heavy metals was further reduced. The leaching concentration of heavy metal Ni in the solidified soil with the solidifying agent content of 6% is 0.01 mg/L ~ 5.03 mg/L, its minimum leaching concentration has met the heavy metal Ni limit specified in Class IV water.

It can be seen from the figure that with the increase of curing age, the leaching concentration of heavy metals shows a decreasing trend. Under the condition of low curing agent dosage (4, 6%), the leaching concentration of heavy metals did not change significantly at the initial stage of curing and showed a significant decreasing trend with the curing time. However, the leaching concentration of heavy metals in the solidified soil with high content of BCP (8, 10%) showed a significant decreasing trend in the early stage of curing; with the increase of curing time, the decreasing speed of heavy metal concentration gradually became gentle. This phenomenon further shows that the amount of curing agent will significantly affect the reaction process of curing agent hydration, heavy metal precipitation, and the formation of hydrated calcium sulfate and other products in the solidified soil^27^.

### Change mechanism of environmental geotechnical properties of BCP-solidified soil

1. Acid buffering capacity of solidified soil

**Figure 10:**
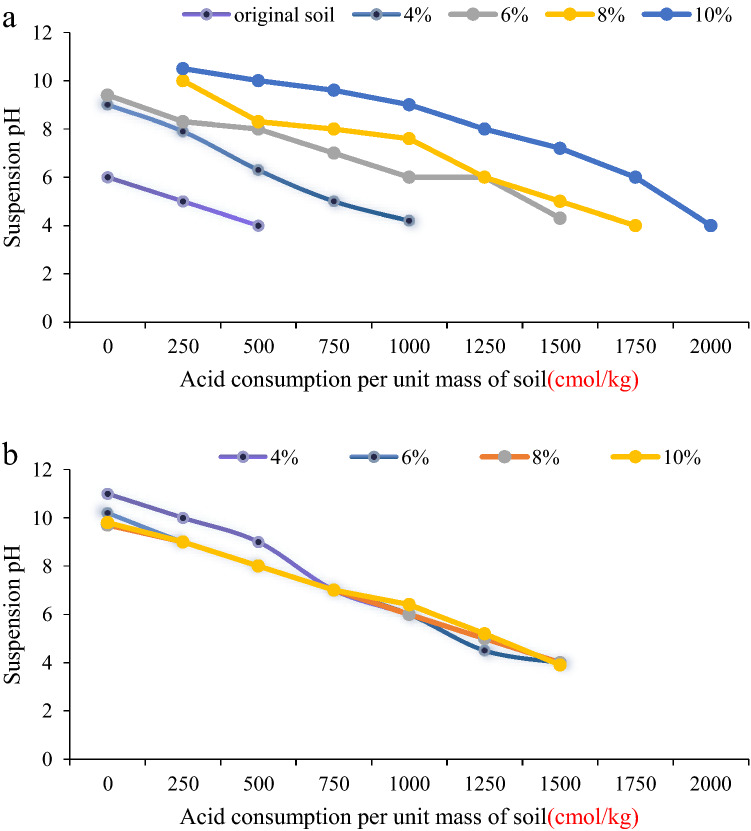
Acid titration curves of soil samples with different contents of curing agent or different curing periods; (**a**) Acid titration curve with curing time of 28 days; (**b**) Acid titration curve with 8% curing agent.

Figures 10a and b describe the effects of curing agent dosage and curing age on acid consumption per unit mass of soil samples, respectively.

It can be seen from Fig. 10a that with the progress of the titration test and the increase of the amount of acid added, the β value of the soil gradually decreases, especially in the mixing stage of the test, the acid buffering capacity decreases sharply. This phenomenon shows that in the mixing stage of the titration test, the dropped H + is mainly neutralized by the OH- in the solidified soil, while in the later stage, the hydration products of the curing agent (C–S–H, AFt and cement stone) and the precipitates of heavy metal hydroxides gradually appeared to neutralize part of the H + . This phenomenon is consistent with the change trend of β values of phosphate-activated steel slag and cement-solidified polluted soil. In addition, with the addition of the curing agent, the change curve of the β value of the contaminated soil gradually shifted upward, indicating that the curing agent effectively increased the acid buffering capacity of the cured soil.

It can be seen from Fig. 10b that with the increase of curing age, the acid buffering capacity of the solidified soil increases gradually. When the pH value of the suspension was 5, the β values calculated from the titration curve were 156.2, 187.6, 201.5, and 219.7 cmol·kg1·pH-1, respectively. The increase of the β value of the solidified soil is mainly because the increase of the curing age promotes the series of reactions in the solidified soil to be complete. The massive accumulation of curing agent hydration products (CSH, AFt, and cement stone), pozzolanic reaction products (CSH), and heavy metal hydroxide precipitates increased the content of alkaline substrates in the suspension, which in turn promoted the improvement of the acid buffering capacity of the solidified soil. Therefore, it can be judged by the change law of acid consumption per unit mass of soil samples and acid buffer coefficient (β value) with the content of curing agent and curing age before and after curing and stabilization, BCP solidification and stabilization can significantly increase the ability of polluted soil to resist external acid erosion, and improve the long-term stability of solidified soil in acid-sensitive environments. When there is an acid environment (acid rain, acid groundwater) erosion in the reused soil of the solidified soil, it can be considered to appropriately increase the dosage of the solidifying agent and the curing time to improve the acid buffering capacity of the solidified soil.

(2) Pore characteristics of solidified soil

The macroscopic strength of the soil is closely related to the microscopic pore characteristics of the soil, so the pore structure characteristics are analyzed to obtain the internal relationship between the two, and to explain the control mechanism of the improvement of the macroscopic strength of the solidified soil. Calculate the pore diameter J with the Washburn formula;

20 $$ {\text{J}} = \frac{{4\mu {\text{cos}}\vartheta }}{{\text{b}}} $$

In the equation, the tension coefficient of the surface is b $\mu$, $\vartheta$ mmm is the contact angle with the soil, usually 139°. b gauge of the mercury pressure.

**Figure 11:**
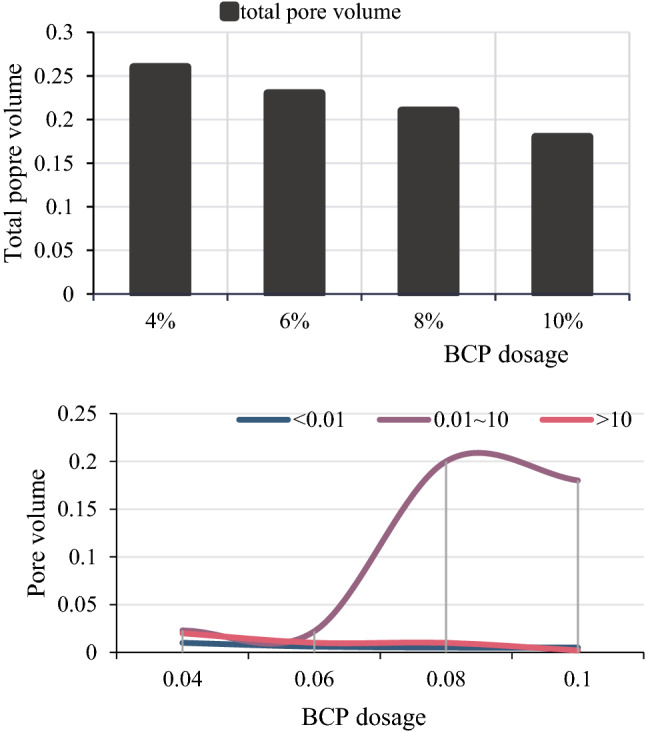
Distribution of pore volume at different curing agent dosages.

The different types of pores in the BCP-solidified soil are divided, as shown in Fig. 11.

It can be seen from Fig. 11 that as the content of the curing agent increases from 4 to 10%, the total pore volume of the cured soil gradually decreases from 0.26 to 0.18 mL/g. The volume of pores and pores in the agglomerates decreased slightly, and the amount of curing agent increased from 4 to 10%, while the corresponding volumes decreased only by 0.006 and 0.017 mL/g. The existing research results show that the interaggregate pores in the soil are an important factor affecting the soil strength. Therefore, it can be seen that the reduction of the pore volume between the aggregates of the BCP-cured stabilized species has made a major contribution to the strength development, and this result is consistent with the change law of the unconfined compressive strength of the solidified soil with the content of the solidifying agent.

(3) Influence mechanism of BCP content on environmental geotechnical properties of solidified soil

After clarifying the effects of curing agent and curing age on the types of products produced by the solidified soil, the quantity of corresponding products was further quantitatively analyzed by the peak intensities of various phases in the XRD analysis. Table 6 lists the intensity changes of the diffraction main peak and the secondary peak of the reaction product in the cured soil with different curing agent contents. It can be found that different reaction products show different changing trends, which are analyzed in detail below. With the increase of curing agent dosage and curing time, the corresponding peak strengths of C–S–H, AFt, and cement stone gradually increased. Therefore, it can be inferred that with the accumulation of the above reaction products in the solidified soil, the soil particle agglomeration effect of the solidified soil is enhanced, and the pores (especially the interparticle pores) are gradually reduced. Therefore, the strength of the contaminated soil increases significantly. On the other hand, with the increase of the above products, the amount of heavy metals stabilized by C–S–H adsorption encapsulation, and AFt ion exchange in the solidified soil increased significantly, which also led to the decrease of the chemical stability, acid buffering capacity and leaching concentration of heavy metals in the solidified soil.

**Table 6:** Diffraction peak intensities of products in cured soil with different BCP contents after curing for 28 days.

Reaction product	Bragg angle	Diffraction intensity		
		8%	20%	50%
Aft	9.1	1035	1120	1470
	15.7	600	720	780
C–S–H	29.5	1108	1330	630
	32	495	550	497
Cement stone	18.2	470	550	500
	34.1	530	555	650
-Ni(0H)_2_	11.4	533	555	630
	22.8	ND	ND	460
-Ni(0H)_2_	19.3	550	570	620
	38.5	ND	ND	370
Ni-FeLDHs(S)	10	680	700	770
	20	490	496	525
Ni-FeLDHs(C)	11.7	ND	ND	760
	23.5	ND	ND	490
Zn_5_(0H)_8_CI2H_2_O	19.1	530	570	640
	38.4	ND	ND	ND
CaZn_2_(0H)_6_2H_2_O	14.3	498	570	600
	25.7	450	515	537

It can be seen from Table 6 that with the increase of the content of the curing agent, the diffraction peaks of the phases containing heavy metals Ni and Zn are gradually enhanced, indicating the gradual increase of their content.

## Conclusions

In this paper, the environmental geotechnical properties of steel slag-based sustainable curing agents (named as BCP curing agent) for repairing nickel-zinc composite heavy metal contaminated clay were systematically investigated, and the control mechanism for the change of environmental geotechnical properties of the contaminated soil was revealed. The main conclusions are as follows: The mixing ratio of the three raw materials of the curing agent is optimized. Taking the unconfined compressive strength of the solidified soil and the stability rate of heavy metals as indicators, the influence rule of factors such as the mass ratio of steel slag to carbide slag, phosphogypsum content, specific surface area of converter steel slag, soluble phosphorus content in phosphogypsum and phosphogypsum pH on the performance of curing agent was found out. The basic soil property parameters of the solidified soil with the curing agent content of 4–10% changed significantly. With the increase of the curing agent, the liquid limit of the contaminated soil gradually decreased, but the engineering classification of the soil remained unchanged, and it was still low liquid limit clay; the specific surface area and organic matter content of the contaminated soil gradually decreased, and the cation exchange capacity and specific gravity gradually increased; the pH value of the leaching solution significantly affects the leaching concentration of heavy metals in the solidified soil. The leaching solution that is too acidic or too basic will promote the increase of the leaching concentration of heavy metals in the solidified soil. The pH value of the leaching solution corresponding to the lowest leaching concentration of heavy metals Ni and Zn in the solidified soil was between 9 and 11. With the increase of the amount of curing agent, the leaching concentration curve of heavy metals changed from "V" type to "U" type, indicating that the ability of the cured soil to resist external acid and alkali erosion gradually increased. The volume of the leaching solution, the mass ratio of soil samples (liquid–solid ratio), and the type of leaching solution significantly affected the leaching concentration of heavy metals in the solidified soil. When the leaching solution was deionized water, the leaching concentration of heavy metals gradually decreased with the increase of liquid–solid ratio; while when the leaching solution was sulfuric acid-nitric acid solution, the leaching concentration of heavy metals gradually increased with the increase of liquid–solid ratio. Compared with the polluted soil, the acid buffering capacity of the solidified soil and the chemical stability of heavy metals have been greatly improved. Multiple linear regression analysis showed that the chemical stability of heavy metals had a more significant effect on the stabilization of heavy metals than the acid buffering capacity of solidified soil. The addition of curing agent significantly changed the pore distribution characteristics of contaminated soil, and the degree of change was significantly affected by the amount of curing agent and the curing age. With the increase of curing agent dosage and curing age, the pore distribution volume and interaggregate pore volume of the cured soil changed most significantly. The amount of curing agent and curing age have an impact on the curing stability effect by changing the quantity and phase morphology of the generated products. In addition, iron ions and chloride ions in polluted soil can promote the stabilization of heavy metals nickel and zinc by forming low-solubility hydroxide precipitation.

## Data Availability

The datasets generated and/or analysed during the current study are not publicly available due [REASON WHY DATA ARE NOT PUBLIC] but are available from the corresponding author on reasonable request.
